# Emerging skeletal growth responses of *Siderastrea siderea* corals to multidecadal anthropogenic impacts in Martinique, Caribbean Sea

**DOI:** 10.1038/s41598-025-08709-5

**Published:** 2025-07-02

**Authors:** Gabriel O. Cardoso, Diego K. Kersting, Thomas C. Brachert, Georg A. Heiss, Reinhold Leinfelder, Jean-Philippe Maréchal, Juan Pablo D’Olivo

**Affiliations:** 1 Institute of Geological Sciences, Freie Universität Berlin, Berlin, Germany; 2 Instituto de Acuicultura de Torre de la Sal, Consejo Superior de Investigaciones Científicas, Ribera de Cabanes, Spain; 3 Institute of Earth System Science and Remote Sensing, Leipzig University, Leipzig, Germany; 4 Reef Check e.V, Bremen, Germany; 5 Nova Blue Environment, Schoelcher, Martinique; 6 Instituto de Ciencias del Mar y Limnología, Unidad Académica de Sistemas Arrecifales, Universidad Nacional Autónoma de México, Puerto Morelos, Mexico

**Keywords:** Coral reefs, Local stressors, Climate change, Sublethal effects, Caribbean sea, Sclerochronology, Ecology, Tropical ecology, Ocean sciences, Climate-change ecology, Ecophysiology

## Abstract

Anthropogenic stressors on Caribbean reefs have shifted coral dominance toward stress-tolerant species such as *Siderastrea siderea*, increasing their importance for reef construction. However, the sublethal responses of these corals to past stressors remain understudied, limiting our understanding of their long-term acclimatization capacity. Here, we reconstructed the annual skeletal growth rates (1912–2020) of twelve *S. siderea* coral colonies from Martinique (Caribbean Sea) and examined their relationships with changes in human population, sea surface temperature (SST), precipitation, river discharge, and agricultural indicators between 1950 and 2020. Long-term growth rate declines observed since the 1950s were initially associated with human population, a proxy for local stress. Warming SST enhanced coral skeletal growth between 1950 and 1985, suggesting optimal temperatures for calcification during this interval. However, skeletal density exhibited a multidecadal (1950–2020) parabolic response, indicating a surpassing of this thermal optimum in recent decades. This parabolic response included persistent skeletal density declines from 2010 to 2020, possibly related to cumulative thermal stress or the interaction with ocean acidification effects. Our findings suggest a limited acclimatization capacity of long-lived *S. siderea* corals to cumulative pressures in Martinique and highlight their vulnerability to the compounding effects of local stressors and climate change.

**Supplementary Information:**

The online version contains supplementary material available at 10.1038/s41598-025-08709-5.

## Introduction

Coral reefs are the most biodiverse marine ecosystems and provide goods and services for millions of people^1,2^. Maintaining elevated habitat complexity is crucial for reef functioning and is contingent on the ability of scleractinian corals to deposit calcium carbonate skeletons^3^. However, human-induced increases in atmospheric greenhouse gases are driving ocean warming and ocean acidification (OA), which, in combination with local stressors, such as overfishing, diseases, and coastal pollution are reducing coral cover and calcification rates worldwide^4–9^. In the Caribbean, coral cover declines began in the 1960s due to local human disturbances and were exacerbated by coral bleaching events triggered by recent thermal anomalies^10,11^, totaling losses of up to 80% since the 1970s^9^. These stressors have led to the demise of fast-growing *Acropora* spp. corals and increased the abundance of stress-tolerant species like *Siderastrea* siderea^10^, thereby increasing their relative contribution to reef carbonate accretion rates^12^. Despite its resilience, *S. siderea* was recently listed as critically endangered on the IUCN Red List due to population declines from cumulative impacts^13^. Understanding how these once resilient and now endangered corals have responded to past changes in environmental conditions is therefore key for projecting how reefs will respond to changing conditions.

Coral growth is sensitive to changes in environmental conditions and relies on the energy supplied by symbionts (Symbiodiniaceae)^14^. Ocean acidification (OA) can affect growth rates by reducing seawater aragonite saturation states (Ω_ar_), which increases metabolic costs for corals to maintain optimal conditions for calcification in their calcifying fluid^15,16^. Ocean warming enhances photosynthesis, respiration, and calcification up to a thermal optimum, after which biological performance decreases and corals face potential sublethal effects (e.g., growth declines), bleaching, or mortality^14,17^. This parabolic thermal performance has been experimentally described^17–20^ and can be influenced by chronic local stressors, such as elevated sedimentation, nutrification, and decreased light due to turbidity^21–24^. Local stressors alone can impact coral photosynthetic yields and increase metabolic costs to produce mucus and actively remove particles^25,26^, ultimately reducing skeletal growth rates. However, the scarcity of long-term, direct growth observations limits our capacity to identify coral thermal thresholds and to examine their relationships (and potential synergies) with past changes in local stressors and climate.

Massive corals typically produce seasonal growth bands visible through X-radiography. These bands offer a record of past growth rates, providing valuable insights into how environmental changes influence coral health over decades to centuries^8,27^. The massive, reef-building coral *S. siderea* is ubiquitous in shallow water reefs in the Atlantic Ocean and is particularly suitable for growth reconstructions due to its relatively large size, slow growth rates (0.2–0.8 cm year^− 1^), and long lifespan (up to centuries)^28^. Previous experiments have demonstrated impressive survival rates of *Siderastrea* corals under different OA, warming, and sedimentation treatments^18–20,29–31^. Nevertheless, long-term growth reductions associated with increases in human population, coastal activities or warming have been observed in colonies from Belize^32,33^, Florida^34^, and Panama^35^. *Siderastrea* corals from other Caribbean regions, including the Lesser Antilles, have experienced more frequent thermal stress^36^, but similar growth impacts remain unknown.

Martinique is part of the Lesser Antilles and shares a history of significant changes with many Caribbean islands, particularly in terms of human population growth, agriculture, and land and water uses^9,37,38^. Coastal eutrophication was the major stressor impacting reefs until the early 21st century^39^, when bleaching-related mortality increased^40,41^. While the accumulation of impacts on the reefs of Martinique is driving phase shifts towards alternate stable states (e.g., macroalgae-dominated communities)^9^, assessing their sublethal effects on individual corals may provide insights into broader population changes^42^. Therefore, to investigate the sublethal effects on *S. siderea* corals and evaluate their ability to acclimatize to long-term changes in environmental conditions, we used annual skeletal growth records obtained from twelve corals collected across four sites in southern Martinique. Specifically, we reconstructed the linear extension, skeletal density, and calcification rates of these corals and investigated their response to past changes in human population, sea surface temperature, precipitation, river discharge, and agricultural indicators between 1950 and 2020.

## Materials and methods

### Historical changes in Martinique

Martinique is a volcanic island characterized by elevated annual temperatures (annual mean 25.5 ± 1.5 °C), abundant precipitation (2025 ± 866 mm year^− 1^), and strong winds (20–55 km h^− 1^)^37^. These favourable climatic conditions for crop growth drove significant land use changes on the island^43^. From the end of the 17th century to the mid-19th century, sugarcane dominated agriculture, driving extensive land clearing^37^. However, sugarcane production decreased because of slavery abolition, decreases in the sugar price, and a shift to polycultures like banana and pineapple^37^. Urban expansion further degraded ecosystems by damming water courses, reducing forest area and mangroves, and impoverishing soils^37^. Most of these changes occurred in the absence of a wastewater treatment system^39^. By 2019, 42.5% of the population was connected to a wastewater treatment system, and 33% of treatment plants met French legal standards, leading to effective treatment of only 20% (Observatoire de l’Eau Martinique, https://www.observatoire-eau-martinique.fr). These land use changes, in parallel with the increased exploitation of wetlands and tourism, have amplified pollution reaching the reefs in Martinique^44^, particularly in the South^38^. At sea, overfishing has long depleted herbivorous fish populations (e.g., Acanthuridae and Scaridae)^45^.

### Sample collection and Preparation

**Fig. 1:**
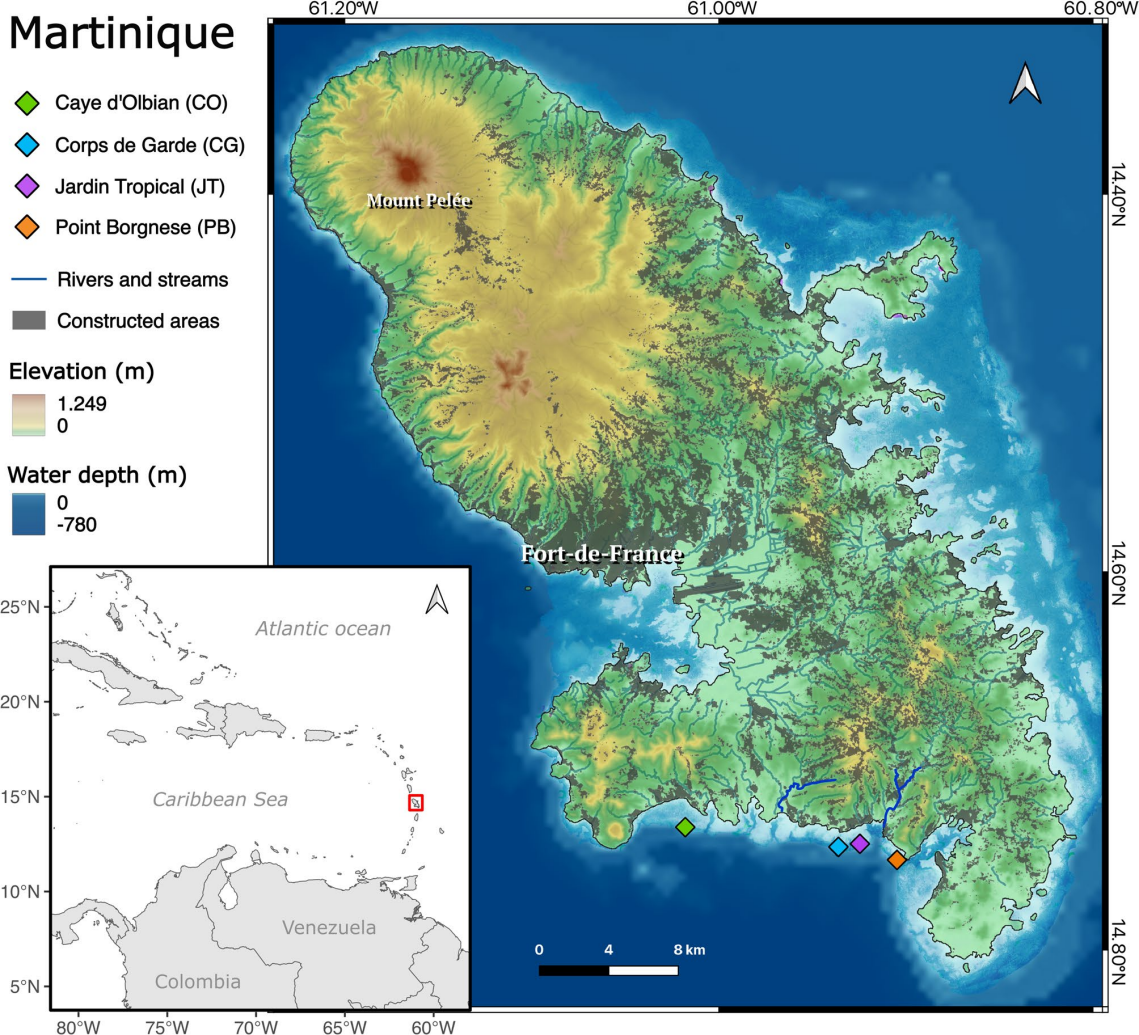
Map of Martinique, Caribbean Sea. Location of collection sites is shown in coloured diamonds. Elevation, water depth, constructed areas (Geoservices 2022, https://geoservices.ign.fr/), and rivers and streams are shown. Major rivers (in terms of volume) discharging in the south are highlighted with bold, dark blue: Oman River and Rivière-Pilote River (from left to right).

Between October and November 2021, twelve coral cores were collected from living colonies of the massive coral *S. siderea* by SCUBA divers using a pneumatic drill (Stanley drill 160189XSTN, 2000 RPM, maximum air pressure of 6 bar/87 PSI, air consumption of 170 L min^-1^) across four sites in southern Martinique (Fig. 1; Table S1). The cores, which were 4 cm in diameter and between 13 and 58 cm in length, were extracted from colonies with a maximum diameter of 50–90 cm living between depths of 4 and 13 m. Concrete plugs with similar diameter to that of the drilled holes were inserted into the holes to minimize bioerosion and promote a faster recovery.

Coral cores were transported to the laboratories of the Institut für Geologische Wissenschaften (Freie Universität Berlin) where they were sliced into slabs with a water-cooled diamond saw blade. Specifically, the Diamond Scheppach saw HSM3500 (230 V ~/50 Hz) manufactured in 2017 was used with a diamond blade of ⌀ 350 × 25.4 × 3 × 10 mm (blade diameter x arbor hole x blade thickness x segment height) and maximum 4300 RPM to produce coral slabs. An in-house made plastic guide was used to ensure a precise cut direction. The surface of the slabs was milled to an even 6 mm thickness using a Tungsten carbide coated drill bit (Dremel 2615993632, ⌀ 19 mm) at the Institut für Erdsystemforschung und Fernerkundung (Universität Leipzig). The slabs were then rinsed and cleaned 4 times in an ultrasonic bath with MQ water and dried at 40 °C for 24 h.

### Data acquisition and processing

To enable the identification of high- and low-density growth bands, the coral slabs were X-rayed (50 kV, 200 mA, exp 60 s) at the Institut für Erdsystemforschung und Fernerkundung (Universität Leipzig) using a digital X-ray cabinet (SHR 50 V)^46^. In *Siderastrea* corals, low-density bands have been described to occur during boreal winter (December – May) and high-density bands during boreal summer (June – November)^28^. It has been argued that sex can affect the timing of band formation^47^. We did not assess sex, but the most recent band in the samples studied here corresponded to a high-density band (Fig. S1), thereby suggesting that they have been formed during the summer (June – November).

The pairs of annual density bands identified in the X-ray images were used to establish a chronology for each coral core and measure their growth parameters^48^. All X-ray images were processed with the aid of the software “Coral X-radiograph Densitometry System” (Coral XDS)^49^. Measured growth parameters included linear extension rates (cm year^− 1^) and skeletal density (g cm^− 3^). Calcification rates (g cm^− 2^ year^− 1^) derived from the product of linear extension and skeletal density. Linear extension is defined as the vertical accretion of the skeleton measured in each pair of high- and low-density bands. Using Coral-XDS, linear transects were drawn perpendicularly to the growth bands, requiring the use of multiple transects to construct one long master chronology for each coral (Fig. S1). To avoid the introduction of artifacts when transitioning between one transect to another (e.g., incomplete bands), overlaps between transects of at least one pair of bands were performed (Fig. S1). Skeletal bulk density (hereafter called skeletal density) measurements were based on grayscale values (luminance) of the X-ray images, which correspond to changes in skeletal density. Coral luminance values were converted to absolute skeletal density values based on the linear relationship determined using a standard material of known density^49^. In this procedure, 6 mm thick aluminium plates and a 9.9 cm long aluminium wedge with a slope of 0.138º and density of 2.71 g cm^− 3^ were X-rayed. In the X-ray image of the wedge, a transect was made along its length to record the gradual changes in density and produce a calibration curve to be used as a density standard. This calibration curve was validated using zero density (air) and a slab of pure aragonite from a *Tridacna maxima* shell of known density (2.93 g cm^− 3^), with a thickness equivalent to that of the corals analyzed^46^. The X-radiographs of aluminium plates were used as background images to correct the images for variations in the X-ray beam intensity and minimize uncertainties in the skeletal density estimations^49^.

Annual linear extension rates were determined by summing the length of each pair of low- and high-density bands. Annual skeletal density values were calculated as the average for each pair of low- and high-density bands. The products of annual linear extension and annual skeletal density were used to determine annual calcification rates. The year 2021 was excluded from the data analysis to avoid measuring incomplete bands, as the last band was still being formed at the time of core extraction.

Master chronologies of linear extension rates, skeletal density, and calcification rates were constructed from coral cores spanning the period from 1917 to 2020. A normalization method (e.g., D’Olivo et al.^50^) was employed to standardize the data over a common period. Specifically, the mean of a growth parameter for each coral record was calculated for two periods: 1993 to 2020, common to 10 out of 12 cores, and 1954 to 2020, common to half the cores. To avoid potential biases from shorter records, the average values of PB02 (1997–2020) and PB04 (2006–2020) were excluded from the normalization process but were still included in the master chronologies. The normalization process involved subtracting the core-specific mean values from each annual raw data point of the corresponding cores. Subsequently, the average mean value of all cores over the period 1954–2020 was added to the cores CO01, CO02, CG01, CG02, CG03, JT01, and JT02. For the cores PB01, PB02, PB03, PB04, and CO03, the average mean value of all cores over the period 1993–2020 was added. This procedure was chosen to reduce the effects of combining records of varying lengths and mean values while preserving the original units. In addition, this procedure reduces potential effects associated with varying depths.

### Environmental parameters

Monthly sea surface temperature (SST) covering the period between 1912 and 2020 centered at 14°N and 60°W was obtained from COADS extended reconstructed SST NOAA NCDCERSSTv5 (ERSSTv5) with a spatial resolution of 2° × 2°^51^ (iridl.ldeo.columbia.edu, accessed in 2023). Total human population in Martinique (counted once a year) from 1950 to 2020 was gathered from the United Nations Population Division (https://population.un.org/wpp/, accessed in 2023). Annual agricultural land data (1000 ha) from 1961 to 2020 and annual sugarcane and banana production (tons) data from 1961 to 2006 were sourced for Martinique from FAO (https://www.fao.org/faostat, accessed in 2023). Monthly river discharge (m^3^ s^− 1^) of the Oman (1995–2020) and Madeleine (2012–2020) rivers, measured at the Dormante (14°29’09.5"N, 60°57’41.7"W) and Point Madeleine (14°29’46.1"N, 60°54’17.0"W) stations, respectively, was obtained from the French Ministry of Ecological Transition, Hydro Portal (www.hydro.eaufrance.fr, accessed in 2023). In situ monthly precipitation data from 1969 to 2021 were collected from the STE Luce station (14°29’25’’ N, 60°58’46” W), available on the Météo-France platform (https://publitheque.meteo.fr/, accessed in 2023). Over the common period of 2012–2020, monthly precipitation at STE Luce is strongly correlated with discharge from both the Oman (*r* = 0.77, *p* < 0.001, *n* = 108) and Madeleine (*r* = 0.77, *p* < 0.001, *n* = 108) rivers (Fig. S2), suggesting that the precipitation data could serve as a proxy for past river discharge.

The precipitation record from STE Luce (1969–2021) was expanded using monthly precipitation (mm) at Fort de France (14°36’00.0’’N, 61°06’00.0’’W) obtained from 1932 to 2018 from the NOAA NCDC (GHCNv2)^52^, available in the KNMI Climate Explorer (https://climexp.knmi.nl, accessed in 2023). The overlap (1993–2018) between the precipitation datasets from STE Luce and NOAA was strongly correlated (*r* = 0.84, *p* < 0.001, *n* = 284; Fig. S2), but owing to its central location in Martinique, Fort-de-France presented a higher mean precipitation. Since STE Luce is more representative of the southern reef sites, the data comprising the period from 1932 to 1968 were rescaled. A detailed description of the rescaling procedure and the application of a Monte Carlo uncertainty estimation for this rescaling is provided in the Supplementary Material (Fig. S3).

Annual averages of all the environmental parameters were calculated to assess their associations with annual coral growth. Minimum and maximum monthly mean sea surface temperatures (SST_min_ and SST_max_) were also calculated to explore growth responses to thermal stress^27^. Environmental parameters were acquired either spanning the longest period available or covering the period of coral growth (1950–2020).

### Statistical analyses

A sequential t-test analysis of regime shifts^53^ was used to detect significant (*p* < 0.05) long-term shifts in the mean of log-transformed growth parameters. To define the minimum duration of a regime, a 10-year cut-off length was determined (i.e., regime shifts should not be shorter than 10 years). In addition, linear regressions and Pearson correlations were performed to identify significant (*p* < 0.05) associations between log-transformed growth parameters and human population, temperature, precipitation, river discharge, and agriculture indicators^27^. To verify these associations, two intervals of equal length (1950–1985 and 1986–2020) were defined based on shifts in calcification rates, and the availability of coral records (*n* ≥ 5) and environmental data (e.g., human population). All analyses were carried out in R using the “corrplot”, “Hmisc”, “ggpmisc”, and “rshift” packages^54^.

## Results

### Coral growth records

Seasonal growth bands were visible in X-radiographs from all the coral cores (Fig. S1). The number of years identified in each core ranged from 14 (2006–2020, PB04) to 108 (1912–2020, JT02) (Table 1). The long-term (1912–2020) mean (± SD) annual linear extension rate based on the normalized data from all twelve coral records is 0.45 ± 0.08 cm, which is consistent with the mean rates (0.43 ± 0.18 cm) reported in previous *S. siderea* records^28,34,47^ (Fig. S4). In contrast, the mean annual skeletal density is 2.01 ± 0.06 g cm^− 3^ and the mean annual calcification is 0.89 ± 0.16 g cm^− 2^, which are higher values than those reported for *S. siderea* in Florida (1.34–1.49 g cm^− 3^ and 0.48–0.57 g cm^− 2^)^34^ and Mexico (1.3–1.5 g cm^− 3^ and 0.32–0.38 g cm^− 2^)^47^. The relationship between growth parameters over the 108-year period shows a strong positive correlation between linear extension and calcification rates (*r* = 0.98, *p* < 0.001, *n* = 108). Weaker positive correlations exist between skeletal density and calcification rates (*r* = 0.39, *p* < 0.001, *n* = 108) and between skeletal density and linear extension rates (*r* = 0.21, *p* = 0.02, *n* = 108).

**Table 1:** Coral core information (collection site, depth, and period assessed) and their corresponding growth characteristics: mean annual (± SD) linear extension rate, skeletal density, and calcification rate.

Core ID	Reef site	Depth	Period	*N* of years	Linear extension rate (cm year^− 1^)	Skeletal density (g cm^− 3^)	Calcification rate (g cm^− 2^ year^− 1^)
CO01	Caye d’Olbian	9.3	1940–2020	80	0.36 ± 0.10	2.12 ± 0.12	0.76 ± 0.21
CO02	Caye d’Olbian	9.6	1962–2020	58	0.42 ± 0.01	2.12 ± 0.13	0.89 ± 0.21
CO03	Caye d’Olbian	13.2	1989–2020	31	0.39 ± 0.08	1.98 ± 0.08	0.77 ± 0.14
CG01	Corps de Garde	7.3	1954–2020	66	0.44 ± 0.01	1.92 ± 0.07	0.85 ± 0.17
CG02	Corps de Garde	7.3	1950–2020	70	0.33 ± 0.06	2.05 ± 0.09	0.67 ± 0.13
CG03	Corps de Garde	6.9	1917–2020	103	0.53 ± 0.14	1.57 ± 0.05	0.84 ± 0.22
JT01	Jardin Tropical	7.3	1948–2020	72	0.40 ± 0.09	1.95 ± 0.11	0.78 ± 0.19
JT02	Jardin Tropical	7.6	1912–2020	108	0.43 ± 0.17	1.90 ± 0.11	0.83 ± 0.33
PB01	Point Borgnèse	9	1993–2020	27	0.45 ± 0.01	1.87 ± 0.06	0.84 ± 0.22
PB02	Point Borgnèse	9.5	1997–2020	23	0.40 ± 0.11	2.05 ± 0.08	0.81 ± 0.23
PB03	Point Borgnèse	4.4	1993–2020	27	0.42 ± 0.11	2.26 ± 0.14	0.96 ± 0.28
PB04	Point Borgnèse	4.5	2006–2020	14	0.39 ± 0.15	2.04 ± 0.16	0.81 ± 0.33

**Fig. 2:**
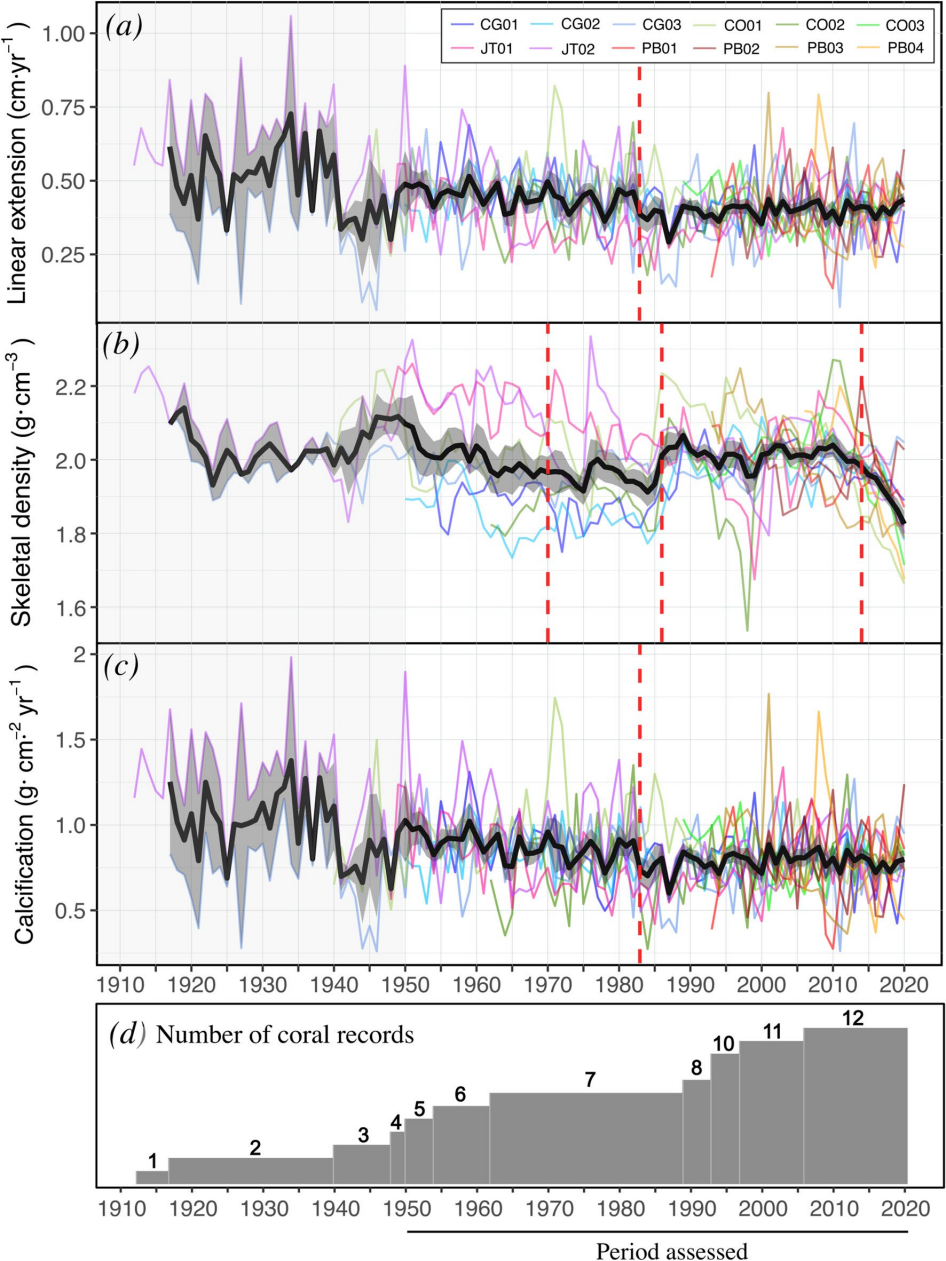
Normalized annual records (1917–2020) of (**a**) linear extension rate, (**b**) skeletal density, and (**c**) calcification rate for coral cores collected in Corps de Garde (CG01-03), Caye d’Olbian (CO01-03), Jardin Tropical (JT01-02), and Pointe Borgnèse (PB01-04). The coloured lines represent individual core records, the black bold lines represent the master chronology, and the envelopes represent the associated standard error. Vertical dashed red lines highlight the timings of significant regime shifts in the mean values. (**d**) The bar chart in the bottom panel shows the number of coral cores available. The period from 1950 to 2020 encompasses the timeframe used to explore growth responses to variations in environmental conditions.

The growth rates from the two longest coral records (JT02 and CG03) showed contrasting temporal trends between 1917 and 2020 (Fig. 2; Fig. S5). While long-term growth declines were found in JT02, CG03 was characterized by overall stable growth with episodic decreases in extension rate during the early-1940s and late 1980s. To improve the statistical sensitivity and interpretability of the results, this study focuses on the period between 1950 and 2020, which includes at least 5 out of the 12 coral records (Fig. 2d).

### Long-term changes in growth rates

Between 1950 and 2020, long-term declines were observed in linear extension (− 0.0024 cm year^− 1^, *r*^2^ = 0.25, *p* < 0.001) and calcification rates (− 0.0026 cm^− 2^ year^− 1^, *r*^2^ = 0.29, *p* < 0.001), whereas skeletal density was characterized by multidecadal fluctuations and comparatively minor declines (-0.0003 g cm^− 3^ year^− 1^, *r*^2^ = 0.05, *p* = 0.02) (Fig. 2). From 1950 to 1985, gradual declines occurred in linear extension rates (− 0.0018 cm year^− 1^, *r*^2^ = 0.23, *p* < 0.01), skeletal density (-0.003 g cm^− 3^ year^− 1^, *r*^2^ = 0.64, *p* < 0.001), and calcification rates (− 0.004 g cm^− 2^ year^− 1^, *r*^2^ = 0.35, *p* < 0.001). Over the most recent decade (2010–2020), a 10.5% decline in skeletal density, consistent across most corals (10 out of 12), was observed. Significant regime shifts toward more negative values were identified in 1983 for linear extension and the calcification rate (Fig. 2). While skeletal density increased three years later (1986), negative shifts were detected in 1962 and 2014.

Decadal averages calculated from annual master chronologies show that all growth parameters shifted from above-average values in 1950–1960 to values below average in 1980–1990 and 2010–2020 (Fig. S6). Linear extension and calcification rates never recovered, despite increases in skeletal density during the following two decades (1990–2000 and 2000–2010).

### Long-term changes in socioenvironmental parameters

**Fig. 3:**
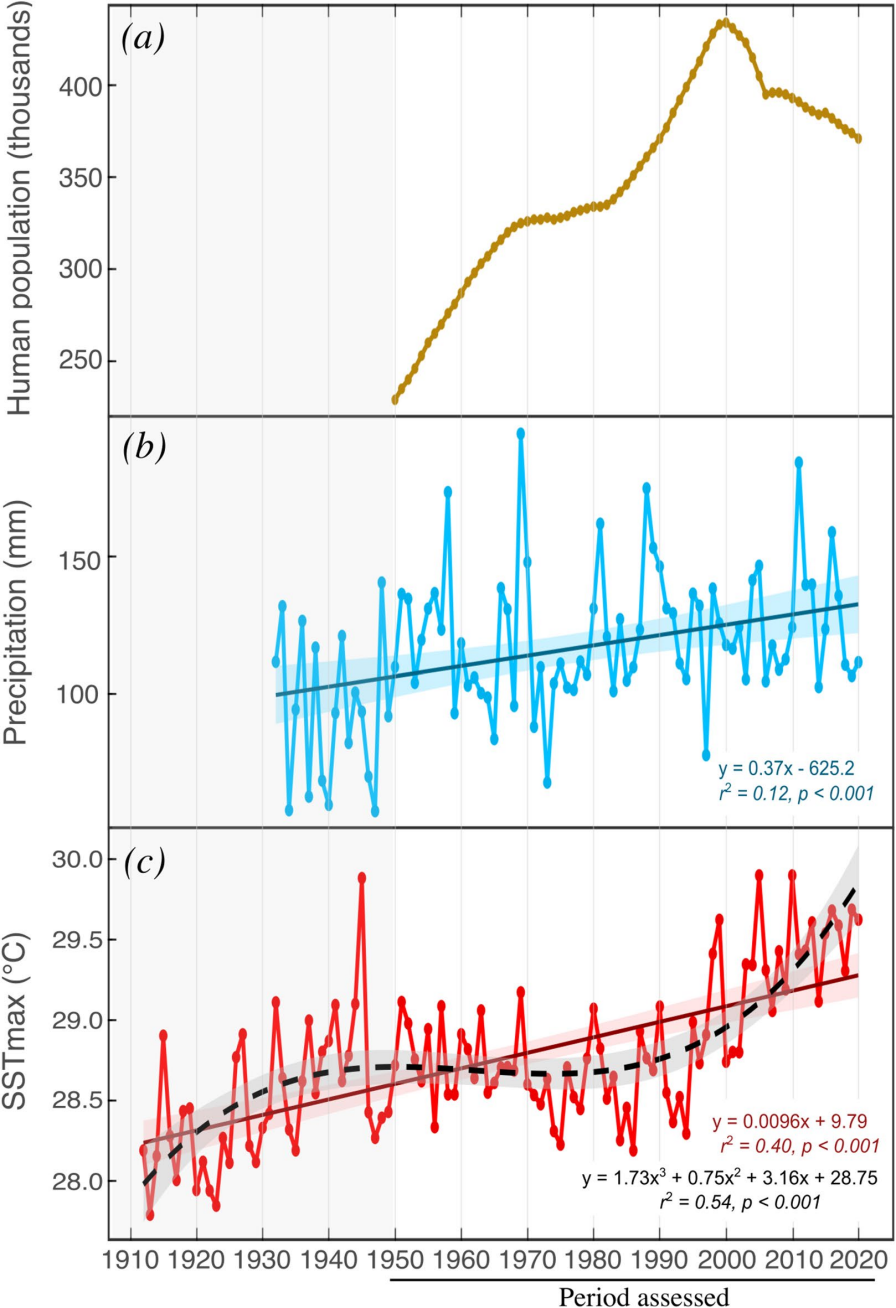
Annual series of (**a**) human population (1950–2020), (**b**) precipitation (1932–2020), and (**c**) monthly mean maximum sea surface temperature (SST_max_; 1912–2020). Linear trend analyses represented by solid lines show significant increases in precipitation (*r*^2^ = 0.06, *p* = 0.01) and SST_max_ (*r*^2^ = 0.40, *p* < 0.001, *n* = 108). A third-degree polynomial (*r*^2^ = 0.54, *p* < 0.001, *n* = 108) is also included for SSTmax, represented by the black dashed line. The envelopes show the associated standard error.

The population of Martinique has experienced a complex long-term trajectory (Fig. 3). The population records document an almost continuous increase between 1950 and 2000, except for temporary stagnation between the 1970s and 1980s. The population nearly doubled from 1950 to 2000, increasing from 229,000 to 434,000 inhabitants. After 2000, following a reverse trend, population gradually decreased to 371,000 by 2020.

Agricultural area followed increases in human population between 1961 and 1985, with declines in sugarcane production (79.8% from 1961 to 1985) being offset by increasing banana production (90% from 1961 to 1978) (Fig. S7). After 1985, human population and agricultural area showed diverging trends, with continued population increases contrasted by declines in agricultural area (except for a 5-year increase around 2010) (Fig. S7). This pattern reflects a broader transition from extensive agriculture to more recent coastal urban expansion (Fig. S8). The clear connection between land use changes and population growth highlights the potential use of population size as a more comprehensive indicator of local stress in Martinique.

Between 1912 and 2020, SST_max_ showed a linear increase of about 0.1 °C per decade (0.01 °C year^− 1^, *r*^2^ = 0.40, *p <* 0.001, *n* = 108), with a stable period observed between the mid 1940s and mid 1990s (Fig. 3). A rapid change in SST_max_ began in the mid-1990s, with mean values between 2000 and 2020 (29.37 ± 0.33 °C) increasing by 0.64 °C compared to the prior two decades (1980–2000, 28.73 ± 0.36 °C). Precipitation showed high interannual variation and a linear increasing trend of 0.37 mm year^− 1^ from 1932 to 2020 (*r*^2^ = 0.06, *p* = 0.01, *n* = 89).

### Socioenvironmental parameters and coral growth

**Fig. 4:**
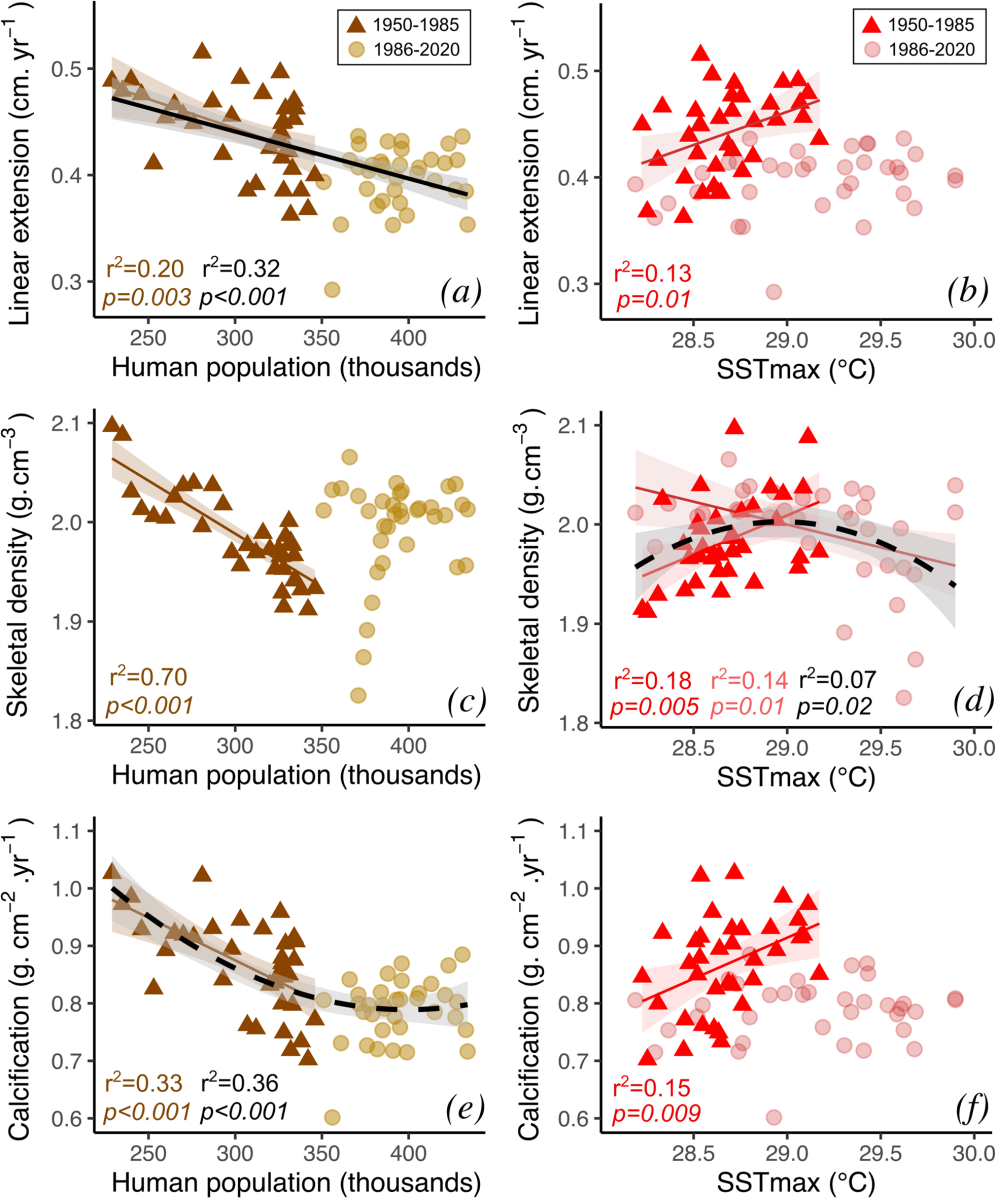
Scatter plots based on log-transformed annual time series of growth parameters and human population (**a**,**c**,**e**) and monthly mean maximum sea surface temperature (SST_max_; **b**,**d**,**f**). Triangles and circles represent the periods 1950 to 1985 and 1986 to 2020, respectively. Regression lines with 95% confidence intervals, and the corresponding coefficients of determination and *p-*values are indicated only when statistical significance (*p* < 0.05) exists. The coloured lines correspond to either 1950 to 1985 or 1986 to 2020, and the black bold lines correspond to 1950 to 2020. The type of regression is represented by the solid (linear) and dashed (polynomial) lines.

Annual coral growth parameters (linear extension, skeletal density, and calcification rate) were compared to temperature (annual mean SST, SST_min,_ and SST_max_), precipitation, human population, river discharge, and agriculture indicators. Between 1950 and 2020, human population shows the strongest association with growth parameters. Over this period, linear extension rate shows a significant negative linear relationship with human population (*r*^2^ = 0.30, *p* < 0.001, *n* = 70), while skeletal density shows no significant relationship (*r*^2^ = 0.01, *p* = 0.16, *n* = 70), and calcification rate shows a significant negative non-linear relationship, better described by a second-degree polynomial (*r*^2^ = 0.36, *p* < 0.001, *n* = 70) (Fig. 4). However, the relationships between all growth parameters and human population (over 1950–2020) are best described when divided into two periods (Fig. 4). The first period (1950–1985) is characterized by negative linear relationships between human population and all three growth parameters (linear extension *r*^2^ = 0.20, *p* = 0.003, *n* = 35; skeletal density *r*^2^ = 0.70, *p* < 0.001, *n* = 35; calcification rate *r*^2^ = 0.33, *p* < 0.001, *n* = 35). These relationships disappear in the second period (1985–2020) (Fig. 4).

Temperature, including the mean SST, SST_min_, and SST_max_, is also significantly correlated with coral growth parameters. The SST_max_ shows the strongest association with coral growth parameters, although with a lower magnitude and a contrasting response compared to human population (Fig. 4; Fig. S9). While local stress is negatively associated to all growth parameters between 1950 and 1985, SST_max_ shows a positive influence on linear extension rate (*r*^2^ = 0.13, *p* = 0.01, *n* = 35), skeletal density (*r*^2^ = 0.18, *p* = 0.005, *n* = 35), and calcification rate (*r*^2^ = 0.15, *p* = 0.009, *n* = 35). However, from 1986 to 2020, linear extension and calcification rates were not related, and skeletal density responded negatively (*r*^2^ = 0.14, *p* = 0.01, *n* = 35; Fig. 4d). This shift in the relationship between SST_max_ and skeletal density over the interval assessed (1950–2020) can be characterized as a parabolic response (*r*^2^ = 0.07, *p* = 0.02, *n* = 70).

Although increases in agricultural area from 1960 to 1985 align temporally with growth reductions, the overall decline from 1961 to 2020 is positively correlated with linear extension (*r* = 0.27, *p* = 0.04, *n* = 60). Similarly, declining sugar production from 1961 to 2006 shows positive correlations with linear extension (*r* = 0.53, *p* < 0.001, *n* = 47), skeletal density (*r* = 0.51, *p* < 0.001, *n* = 47), and calcification rate (*r* = 0.60, *p* < 0.001, *n* = 47). In contrast, increases in banana production are inversely correlated with linear extension (*r* = -0.46, *p* = 0.001, *n* = 47) and calcification (*r* = -0.42, *p* = 0.003, *n* = 47). No significant correlations were found between growth parameters and precipitation or river discharge (Fig. S9). Although this study focuses on a master chronology, it is also worth noticing that no significant (*p* < 0.05) linear relationship was found between coral sclerochronological characteristics and sampling depths (Fig. S10), contrasting with the well-described depth effects on coral metabolism and skeletal structure formation^55,56^.

### Warming and bleaching events

**Fig. 5:**
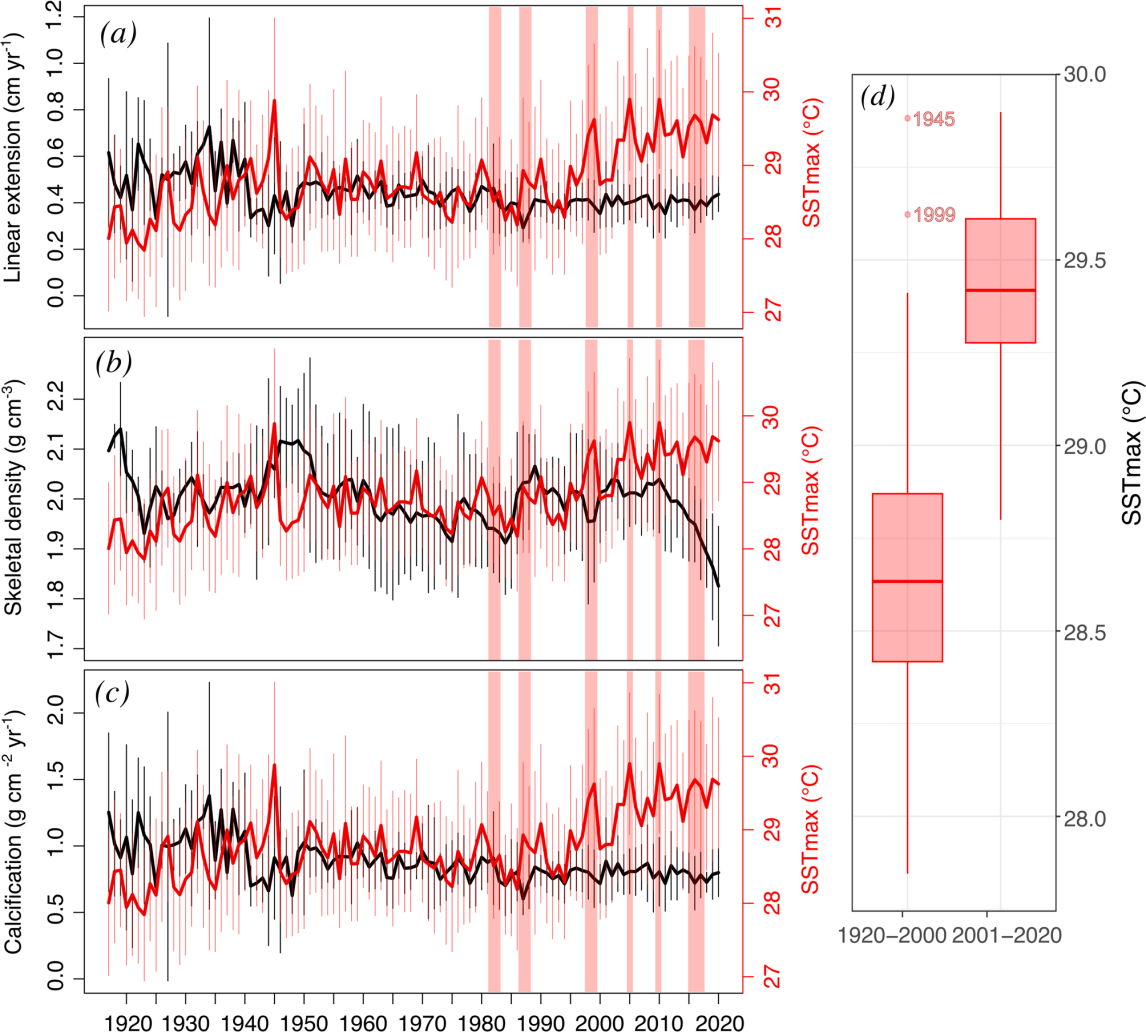
Master chronologies of linear extension rates (**a**), skeletal density (**b**), calcification rates (**c**), and sea surface temperature maximum (SST_max_) from 1917–2020. The vertical lines indicate the standard deviation. The vertical red bars represent mass bleaching events documented in the wider Caribbean (Glynn, 1991; Eakin et al., 2010, 2022; Muñiz-Castillo et al., 2019). Boxplots (**d**) show the SST_max_ values from two periods: 1920–2000 and 2001–2020.

Maximum SST increased quickly during the most recent decades (Fig. 5). For instance, from 1950 to 2020, SST_max_ records registered only seven years of temperatures above 29 °C, most notably during the 1990s (1990, 1998, and 1999). In contrast, SST_max_ surpassed 29 °C every year from 2003 to 2020. Some of these warm periods coincide with documented bleaching events in the Caribbean, including 1982–1983 and 1986–1987^57^, 1997–1998 and 2005^40^, and 2010 and 2015–2017^41^.

Several years of high temperatures and documented mass coral bleaching episodes coincide with short-term decreases in growth. Notably, extension rates decreased in 1986–1987 and both extension rates and skeletal density decreased in 1982–1983 and 1998–1999. Short-lived acute declines in skeletal density around 1998 were observed in the corals CO02, JT01, and JT02. Similarly, sustained high temperatures from 2010 to 2020 coincided with a continuous decrease in skeletal density. However, it is important to note that other years of elevated metabolism and skeletal structure formation temperatures (e.g., 1957, 1963, 1969, 1990) and bleaching events (2005 and 2010) did not correspond to notable changes in growth.

## Discussion

Although the life-history traits of *S. siderea* corals, including massive morphology and generalist symbiont types, have enabled them to withstand a range of stressors and become main contributors to reef carbonate production^10,12^, significant declines in their populations have led to their classification as critically endangered^13^. Consistent with this classification, our results show that the colonies in Martinique have experienced long-term, sublethal effects from local activities since the 1950s, with negative effects of climate change emerging after the 1980s. These findings align with reports from Belize^18,33^ and highlight how corals once considered resilient may have a limited capacity to acclimatize to cumulative warming, shifting from ‘winners’ to ‘losers’ over the long term.

The associations found between coral growth reductions and increased socioenvironmental pressures in Martinique show that chronic stress from local activities preceded the negative effects of climate change. Multidecadal declines in all coral growth parameters (1950–1985) occurred during a period characterized by relatively stable SST values but significant increases in human population and agriculture (Fig. 3, Fig. S7 and Fig. S8). This result provides evidence of a temporal association between sublethal effects in *S. siderea* corals and socioenvironmental changes previously documented as drivers of reef degradation^37–39,44^. Similarly, long-term growth reductions in *S. siderea* corals have been associated with increased coastal sedimentation and eutrophication from the Panama Canal construction^35^ and an increasing human population in Belize^33^. Land-based disturbances also appear to affect skeletal density, as observed here and in *Orbicella annularis* from the Gulf of Mexico^58^. *Siderastrea* corals tolerate high sedimentation levels, including short (1–10 days) burial periods, but chronic, long-lasting sedimentation can cause growth declines or even mortality^30,35,59^.

Despite the lack of a relationship between coral growth and precipitation or river discharge, the potential contribution of sedimentation to the growth reductions observed here cannot be discarded. This result indicates that other factors rather than water volume are more likely to have influenced the observed coral growth patterns (e.g., D’Olivo & McCulloch^60^). For instance, factors associated with light availability are known to influence coral metabolism and mortality (e.g., López-Londoño et al.^24^). In our samples, the lack of relationship between coral sclerochronological characteristics and sampling depths (Fig. S10) contrasts with the well-described depth effects on coral metabolism and skeletal structure formation^55,56^, and might be explained by the limited number of samples and their relatively narrow depth range (< 10 m).

While the long-term changes in seawater quality in Martinique remain poorly understood, the temporal relationship between declining coral growth and increasing population could suggest a contribution from deteriorating water quality. Indeed, coastal pollution from agricultural runoff and wastewater discharge has long been associated with coral cover loss and shifts toward algal-dominated communities in Martinique^9,39,44^. Fertilizers and wastewater are enriched in dissolved inorganic nitrogen (DIN), which alters the N:P stoichiometry in surface waters and promotes algal blooms^7^. Harmful algal blooms have been a major issue in the Caribbean for over five decades^7^ and can reduce coral growth by increasing shading and microbial activity on coral surfaces^5,25,26,61^. Experiments have also shown that enrichment with nitrogen can reduce skeletal density, likely due to coral phosphorus starvation^62^. The long-term growth rate declines observed here point to similar chronic negative effects, particularly before the 1990s. More recently, the stabilization of calcification rates and the lack of evident growth response at the highest population levels might reflect an alteration in the nature of local stressors (e.g., shifting inputs from terrigenous sources) or suggest an increasing influence of temperature. While this stabilization may offer hope for the future of corals in Martinique, it ultimately highlights the urgency of reducing land-based impacts in the context of climate change^5,63^.

Coral growth responses to climate change are multifaceted and involve gradual changes linked to ocean warming, long-term declines from ocean acidification, and episodic reductions from bleaching events^8,22,34^. The responses to long-term warming observed here indicate that temperatures between 1950 and 1985 (mean SST_max_ 28.67 ± 0.24 °C) enhanced metabolic efficiency and enabled the optimal exchange of metabolites necessary for light-enhanced calcification^14^. Calcification is optimal near the coral’s upper thermal limit, and increased calcification rates with 20th -century warming have also been observed in various locations^8^. However, as temperatures continue to rise, corals begin to reach bleaching thresholds, defined as sustained SSTs of 1 °C above the usual summertime maximum^64^. In Martinique, the mean SST_max_ (29.37 ± 0.33 °C) between 2000 and 2020 was 0.7 °C higher than that in period from 1950 to 1985. Skeletal density responded negatively to warming between 1985 and 2020, forming a parabolic response from 1950 to 2020. Although weak (*r*^2^ = 0.07, *p* = 0.02), this parabolic response may indicate a surpassing of the thermal optimal for calcification, as previously proposed in experiments with *S. siderea*^19,20^ and other scleractinian coral species^17,18^.

Skeletal density declines associated with cumulative warming between 2010 and 2020 did not appear to correlate with changes in linear extension or calcification rates. This growth response contrasts with episodic declines in linear extension commonly observed following thermal stress events^22,50,65,66^. Nevertheless, decreases in skeletal density with no changes in linear extension have been observed in *Porites* sp. from the Great Barrier Reef (GBR)^15^ and *S. siderea* corals from Florida^34^ and were attributed to OA. Additionally, *S. siderea* colonies from Mexico transplanted into low Ω_ar_ settings presented reduced skeletal density but no change in linear extension^67^. Alterations in the skeletal architecture of *S. siderea* corals, such as reduced corallite in-filling driven by lower Ω_ar_ levels^29^, may provide a mechanistic explanation for declines in skeletal density. However, the rate of decline in Martinique (10.5% in one decade) starkly contrasts with the gradual reductions reported in Florida (~ 7% from 1878 to 2013) and the GBR (7–18% from 1939 to 2013)^15,34^. The absence of in situ, long-term seawater carbonate chemistry data prevents fully elucidating the relationship between OA and the skeletal density of *S. siderea* corals in Martinique. Nevertheless, their recent sharp decline might suggest a synergistic interaction between stressors.

This hypothesis is supported by the non-linear decline in skeletal density observed here, which aligns with coral responses to the synergy between OA and thermal stress^19,20,68^. Although some experiments suggest a high tolerance of *S. siderea* corals to OA^18,31^, thermal stress can amplify OA effects by lowering coral control over their calcifying fluid (e.g., Guillermic et al.^69^), which is expected to limit skeletal formation by diminishing the pumping of Ca^2−^, removal of H^+^, and conversion of HCO_3_^−^ to CO_3_^2−^^16^. In addition, strategies to cope with heat stress, such as shuffling to more thermally tolerant symbionts, may increase susceptibility to OA^68^. This thermal influence may explain the contrast with the gradual decreases in skeletal density observed in *S. siderea* corals from Florida, where OA effects are more pronounced^70^, but annual temperatures are cooler than those in the Lesser Antilles^34^. The stable calcification rates observed here from 2010 to 2020, despite the pronounced decreased in skeletal density, reflect the predominant influence of extension. Whether maintaining the calcification rate at the expense of skeletal density is a strategy to withstand warming or a broader response to combined stressors deserves further investigation. However, as indicated by our results and those of previous studies^18,20,29,33^, future climate change is likely to further reduce the calcification and survival rates of *S. siderea* corals.

Recurrent bleaching events triggered by climate change are another threat to *S. siderea* corals. The episodic declines in extension rates and skeletal density observed here coincide with the mass events documented in 1982–1983, 1986–1987, and 1998–1999^41,57^. Coral bleaching affects the translocation of metabolites needed for calcification (e.g., dissolved inorganic carbon), causing overall physiological stress and reduced calcification^21^. The level of physiological stress from bleaching is determined by coral susceptibility and health prior to heat stress, and the magnitude and duration of the thermal anomaly^71^. These factors can lead to various bleaching responses, including partial mortality^72^ and reproductive output alterations^71^, which could help explain the lack of growth anomalies associated with regional bleaching events documented in 2005 and 2010^41^. Nevertheless, the effects of the 1982–1983, 1986–1987, and 1998–1999 events are evident in the growth record as episodic declines. Similar growth reductions following the 1998 bleaching event were observed globally, including the Red Sea^65^, Australia^50,66^, and Caribbean reef provinces^22^. In addition, the lack of recovery in calcification rates following 1982–1983 shows prolonged effects from thermal stress. These prolonged effects indicate a reduced capacity to rebound from episodic thermal anomalies or bleaching, likely related to the cumulative thermal and local stressful conditions.

Both local stressors and SST_max_, the latter associated with global-scale patterns of climate change, have influenced the growth of *S. siderea* corals in Martinique. Although the synergistic effects of local and climate stressors on coral reefs are not yet fully understood^73^, previous research has indicated that local stressors can exacerbate the adverse impacts of thermal stress^5,7,23,65^. For example, assuming that coral growth responses are linked to energy allocation and availability^21^, depleted energy reserves due to chronic pollution in Martinique may have increased the sensitivity of *S. siderea* corals to thermal stress and OA. In addition, the occurrence of diseases and hurricanes^9,13^, coupled with consecutive years of suboptimal high temperatures and longer and more frequent marine heat waves, may have compounded the reduction in energy reserves. While this combination of stressors is common in most Caribbean reefs^9^, our findings highlight that a stress-tolerant and resilient coral species is now critically endangered.

The calcification rates observed in Martinique are the highest reported for *S. siderea* corals in the Caribbean, underscoring their importance for reef carbonate production. However, the long-term declines in calcification rates represent relevant losses in reef carbonate accretion, which are projected to be lower than sea-level rise under future climate scenarios^3,4^. Reef structure loss increases the vulnerability of islands and coastal areas to storms, coastal erosion, and sea-level rise^74^. In Martinique, tourism infrastructure (i.e., hotels, shops) located in coastal areas suffers from flooding and erosion^38^, but coral cover is declining due to a combination of overfishing, coastal sedimentation and pollution, bleaching events, diseases, and hurricanes^9^. Changing this scenario will require adaptive management strategies that consider multiple users, recognize the connectivity between ecosystems such as mangroves and coral reefs, and address their common stressors^63,73^. For instance, expanding sewage treatment systems with nutrient removal capabilities could reduce coastal eutrophication and support coral reef recovery^7^, thereby increasing the resilience of mangroves and seagrass beds^73^. In addition, conserving and restoring critical ecological processes in coral reefs, such as herbivory^63^ and reef framework production^3^, may increase the resilience of these ecosystems to climate change impacts^1,75^. Nonetheless, our findings highlight that safeguarding and restoring these unique ecosystems requires localized conservation efforts to be complemented by robust global climate change mitigation.

## Electronic supplementary material

Below is the link to the electronic supplementary material.

Supplementary Material 1

## Data Availability

The datasets generated and analysed during the current study are available in the Zenodo online repository (10.5281/zenodo.15224673) (Cardoso et al., 2025).
