# Supplementary material for: Emerging skeletal growth responses of Siderastrea siderea corals to multidecadal anthropogenic impacts in Martinique, Caribbean Sea
Source: Sci Rep. 2025 Jul 2;15:23127. doi: 10.1038/s41598-025-08709-5 (PMC12222656; doi:10.1038/s41598-025-08709-5)
Supplement: Supplementary file 1 — Supplementary Material 1 [file 41598_2025_8709_MOESM1_ESM.pdf]

Supplementary information for

Emerging skeletal growth responses of *Siderastrea siderea* corals to multidecadal anthropogenic impacts in Martinique, Caribbean Sea

Gabriel O. Cardoso[1*], Diego K. Kersting[2], Thomas C. Brachert[3], Georg A. Heiss[1,4], Reinhold Leinfelder[1], Jean-Philippe Maréchal[5], Juan Pablo D'Olivo[1,6]

1– Institute of Geological Sciences, Freie Universität Berlin, Berlin, DE

2– Instituto de Acuicultura de Torre de la Sal, Consejo Superior de Investigaciones Científicas, Ribera de Cabanes, ES

3– Institute of Earth System Science and Remote Sensing, Leipzig University, Leipzig, DE

4– Reef Check e.V., Bremen, DE

5– Nova Blue Environment, MQ

6– Unidad Académica de Sistemas Arrecifales, Instituto de Ciencias del Mar y Limnología, Universidad Nacional Autónoma de México, Puerto Morelos, Mexico

* Corresponding author: gabrielcardosoeco@gmail.com

Core ID	Reef site	Collection date	Depth (m)	Latitude	Longitude	Colony diameter (cm)	Period	N of years	Linear extension rate ($mm \cdot y^{-1}$)	Skeletal density ($g \cdot cm^{-3}$)	Calcification rate ($g \cdot cm^{-2} \cdot y^{-1}$)
CO-01	Caye d'Olbian	10.29.21	9.3	14°27'53.6"N	61°01'04.7"W	~60	1940-2020	80	3.63 ± 1.00	2.12 ± 0.12	0.77 ± 0.21
CO-02	Caye d'Olbian	11.01.21	9.6	14°27'53.6"N	61°01'04.7"W	~70	1962-2020	58	4.18 ± 0.97	2.12 ± 0.13	0.89 ± 0.21
CO-03	Caye d'Olbian	11.04.21	13.2	14°27'52.5"N	61°01'09.2"W	~40	1989-2020	31	3.93 ± 0.78	1.98 ± 0.08	0.78 ± 0.14
CG-01	Corps de Garde	10.30.21	7.3	14°27'16.0"N	60°56'10.2"W	~70	1954-2020	66	4.44 ± 0.93	1.92 ± 0.07	0.85 ± 0.17
CG-02	Corps de Garde	10.30.21	7.3	14°27'16.0"N	60°56'10.2"W	-	1950-2020	70	3.28 ± 0.66	2.05 ± 0.09	0.67 ± 0.13
CG-03	Corps de Garde	11.02.21	6.9	14°27'16.5"N	60°56'09.0"W	~90	1917-2020	103	5.31 ± 1.41	1.57 ± 0.05	0.84 ± 0.22
JT-01	Jardin Tropical	11.03.21	7.3	14°27'20.2"N	60°55'29.0"W	~90	1948-2020	72	3.99 ± 0.93	1.95 ± 0.11	0.78 ± 0.19
JT-02	Jardin Tropical	11.03.21	7.6	14°27'22.4"N	60°55'28.8"W	~70	1912-2020	108	4.36 ± 1.69	1.90 ± 0.11	0.83 ± 0.33
PB-01	Pointe Borgnèse	10.29.21	9	14°26'51.7"N	60°54'17.3"W	~70	1993-2020	27	4.53 ± 0.12	1.87 ± 0.06	0.84 ± 0.22
PB-02	Pointe Borgnèse	10.31.21	9.5	14°26'51.7"N	60°54'17.3"W	~50	1997-2020	23	3.99 ± 1.15	2.05 ± 0.08	0.81 ± 0.23
PB-03	Pointe Borgnèse	10.31.21	4.4	14°27'00.5"N	60°54'10.7"W	-	1993-2020	27	4.24 ± 1.17	2.26 ± 0.14	0.96 ± 0.28
PB-04	Pointe Borgnèse	10.31.21	4.5	14°26'56.3"N	60°54'09.6"W	~80	2006-2020	14	3.93 ± 1.50	2.04 ± 0.16	0.81 ± 0.33

Table S1. Coral core information (collection site, collection date, depth, coordinates, colony diameter, period assessed, number of growth years assessed) and their corresponding growth characteristics: mean annual (± SD) linear extension rate, skeletal density, and calcification rate.

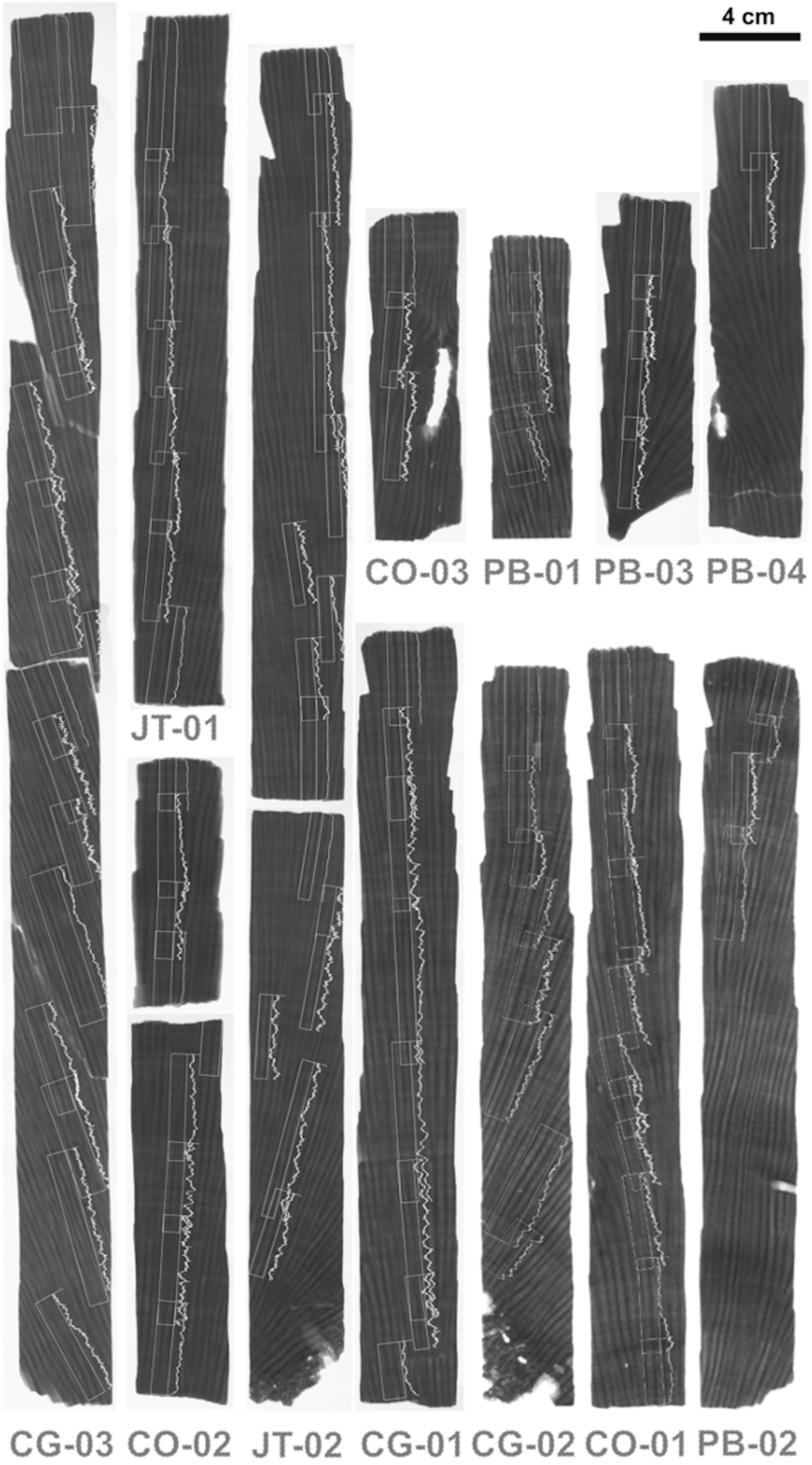

Fig. S1 Composite of X-ray images from *Siderastrea siderea* coral slabs showing density growth bands and the transects made in Coral X-radiograph Densitometry System (Coral XDS[1]) to measure growth parameters. Overlaps between transects of a least one pair of bands were performed to secure measuring the entire length of growth bands. The composite was created to facilitate the appreciation of the growth bands, and was based the original X-rays, used for growth measurements, which did not cover entire length of the cores. Some of the images had their contrast enhanced.

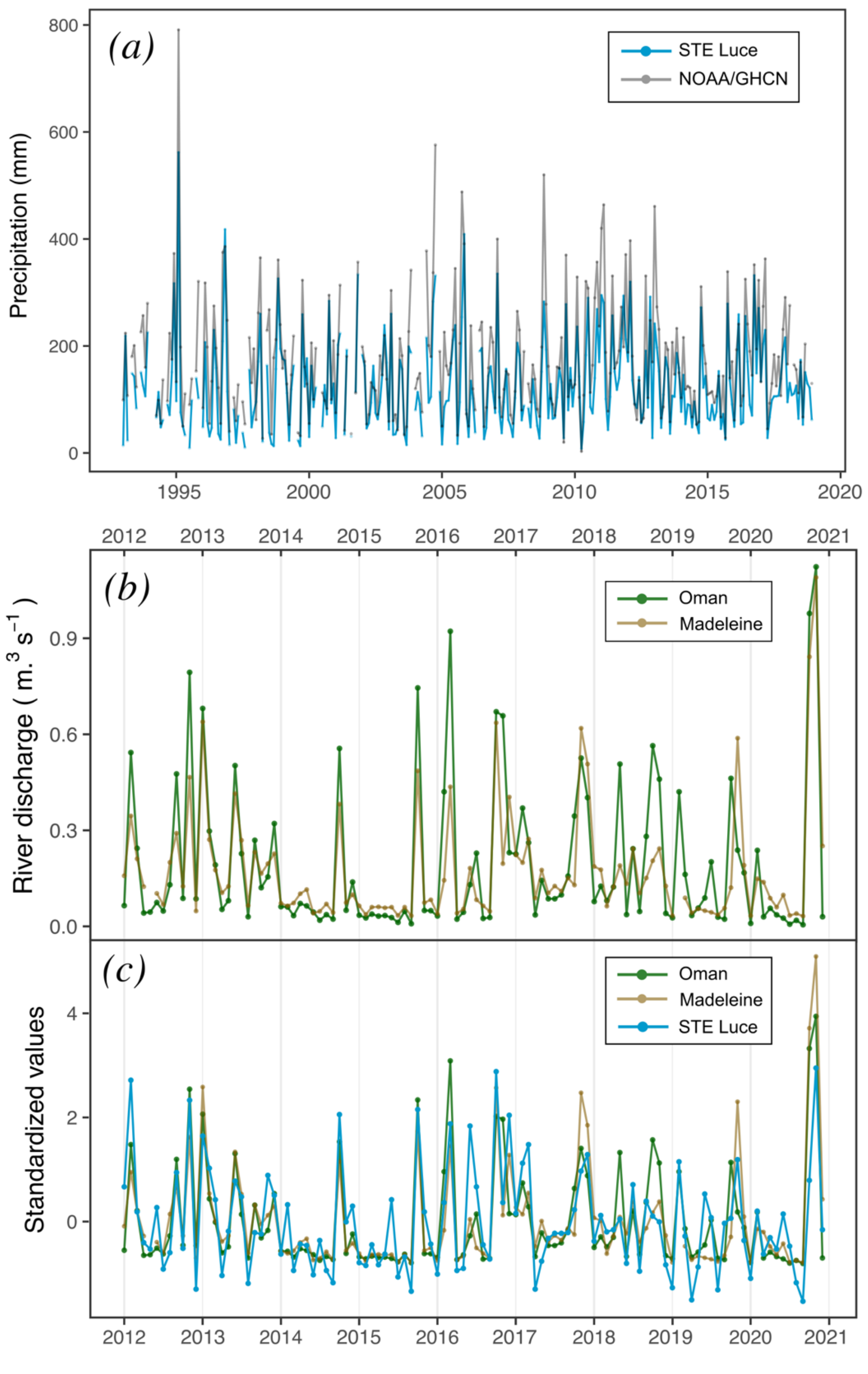

Fig. S2 (a) Monthly precipitation (1993-2018) from STE Luce station (blue) and NOAA/NCDC/GHCN (gray) were strongly correlated ($r = 0.84$, $p < 0.001$, $n = 284$). (b) Monthly river discharge of Oman (2012-2020) and Madeleine (2012-2020), and (c) standardized values of river discharge from Oman and Madeleine rivers and precipitation data from STE Luce station from 2012 to 2020. During this period, Precipitation was significantly correlated with the discharge from Oman ($r = 0.77$, $p < 0.001$, $n = 108$) and Madeleine ($r = 0.77$, $p < 0.001$, $n = 108$).

Precipitation – rescaling procedure and Monte Carlo uncertainty propagation

The overlap (1993-2018) between the precipitation datasets from STE Luce and NOAA was strongly correlated (r = 0.84, p < 0.001, n = 284; Fig. S2a), but due to its central location in Martinique, Fort-de-France presented a higher mean precipitation. Since STE Luce is more representative of the southern reef sites, the data comprising the period from 1932 to 1968 was rescaled. To rescale the data, the mean of each dataset was calculated over the common period of 1993 to 2018 and their difference (49.47 mm) was subtracted from each annual precipitation values from Fort-de-France. A Monte Carlo uncertainty propagation was applied to account for potential biases introduced by this rescaling. First, we calculated the mean difference (49.47 mm) and standard deviation (22.39 mm) between the two datasets over the common period (1993 to 2018). These values were used to generate, for each year, 10,000 simulations of rescaling factors (i.e. precipitation values) randomly drawn from a normal distribution with a mean of 49.47 ± 22.39 mm. These rescaling factors were applied to the gap period (1932–1968) to produce a range of simulated precipitation values, representing the lower and upper bounds of the 90% confidence interval of the rescaled uncertainty (Fig. S3).

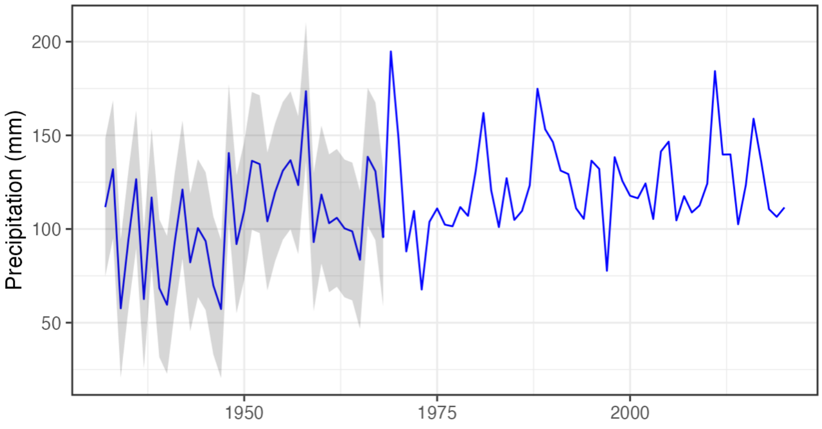

Fig. S3 Annual precipitation values (mm) between 1932 and 2020 from combined STE Luce and Fort-de-France datasets. Gray envelopes show the lower and upper bounds (90% confidence interval) of the rescaled Monte Carlo propagated uncertainty for the expanded period (1932 to 1968).

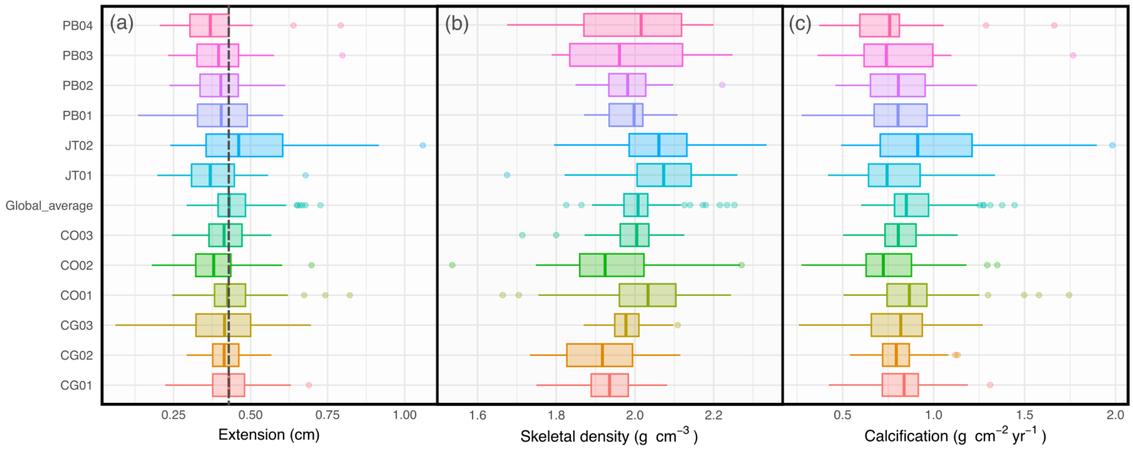

Fig S4. Boxplots showing (a) annual linear extension, (b) skeletal density, and (c) calcification rate data of *Siderastrea siderea* corals (n = 12) from southern Martinique over 1912 to 2020. Global average represents the average of all cores. Vertical dashed line represents the average of annual linear extension rates reported for *S. siderea* in Stearn et al.[2] (n = 2), Hubbard & Scaturo[3] (n = 201), Huston[4] (n = 10), Guzman et al.[5] (n = 50), Guzman & Tudhope[6] (n = 5), Torres & Morelock[7] (n = 5), Carricart-Ganivet et al.[8] (n = 2), DeLong et al.[9] (n = 4), Flannery et al.[10] (n = 1), and Rippe et al.[11] (n = 38).

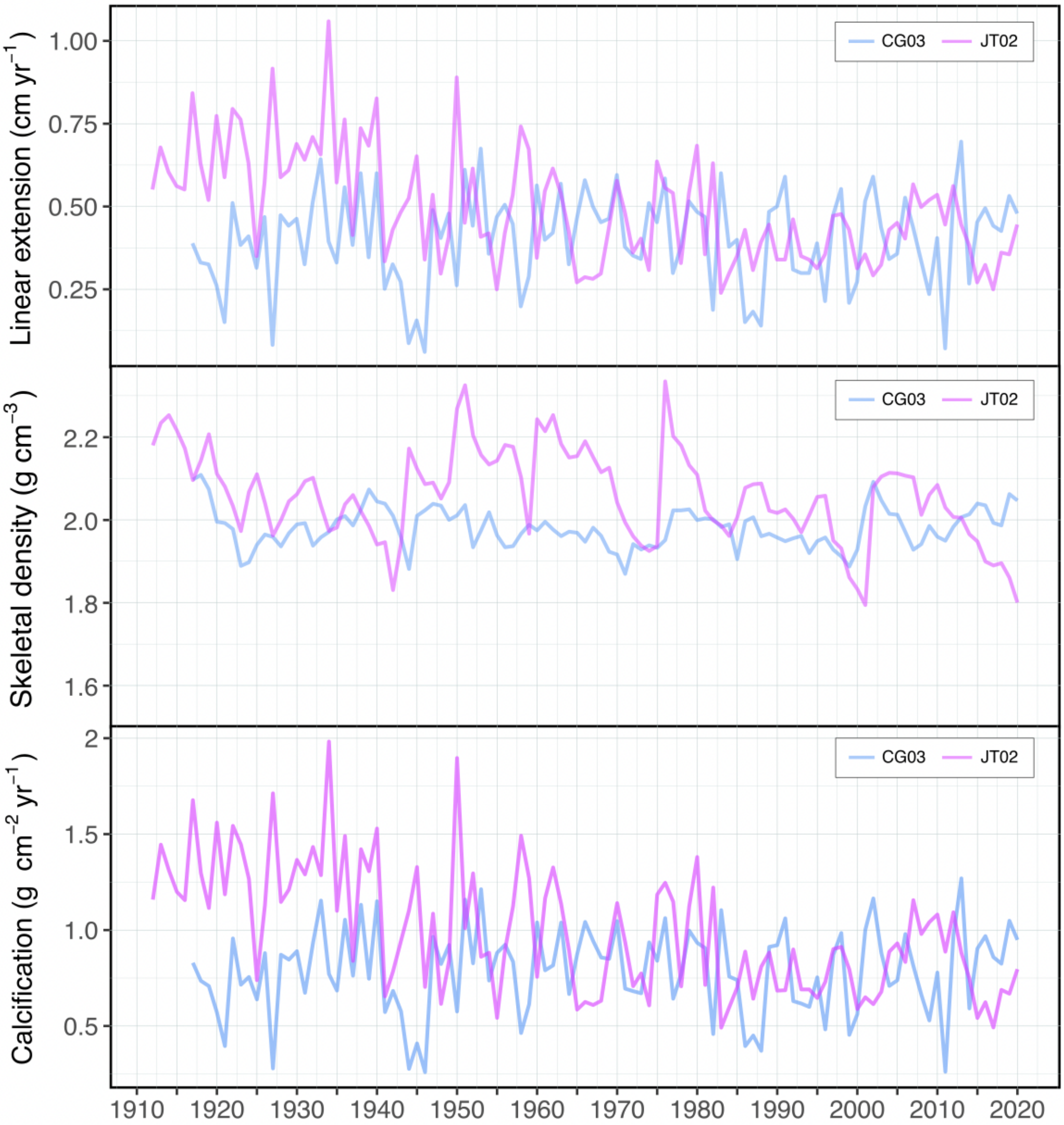

Fig. S5 Annual linear extension rates, skeletal density, and calcification rates for coral cores collected in Corps de Garde (CG03) and Jardin Tropical (JT02) show contrasting temporal trends.

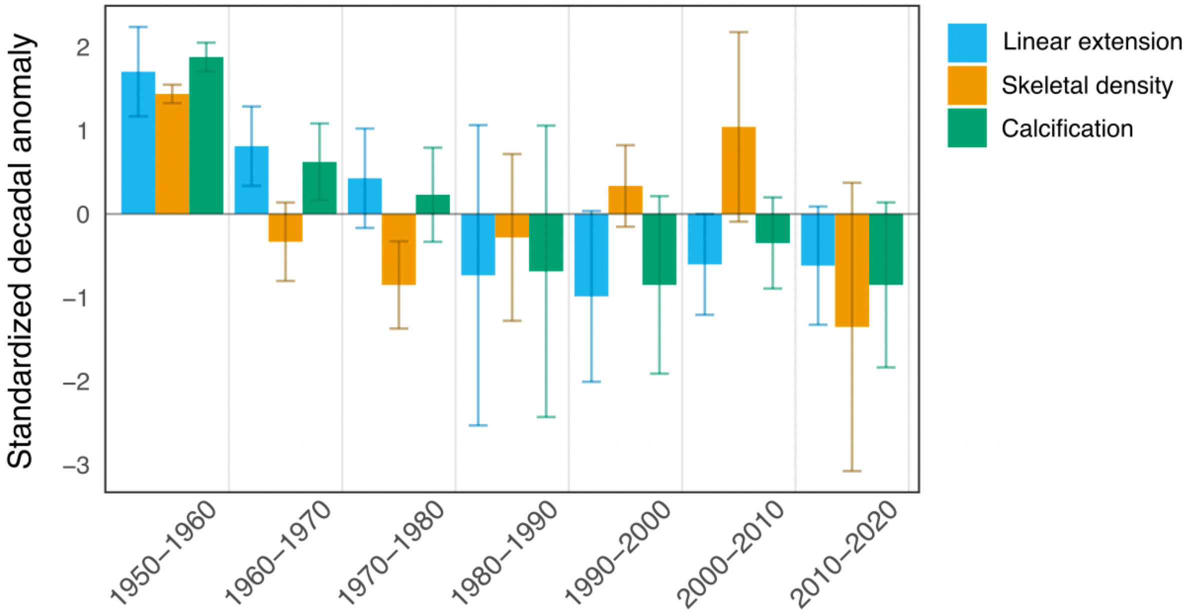

Fig. S6 Standardized anomalies of decadal averages from master chronologies of linear extension (green), skeletal density (yellow), calcification (blue) over 1950 to 2020. Error bars represent the standard error of each decade (n = 10).

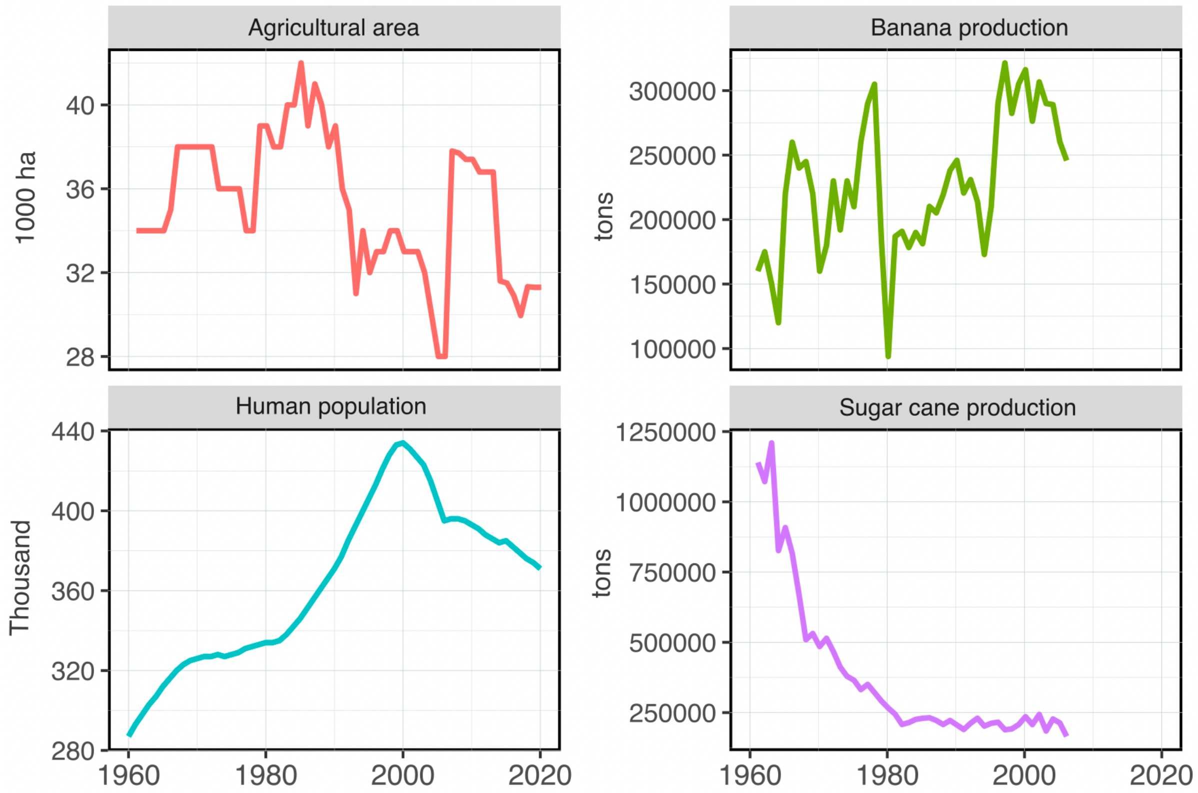

Fig. S7 Main land use changes in Martinique. Agricultural area (1961 to 2020) shows highest values during the early 1980s and a 5-years increase around 2010, but an overall decrease from 1961 to 2020. Human population shows a complex history, with increasing trend from 1960 to 2000, and a decreasing trend afterwards (2000 to 2020). Banana production (1961 to 2006) shows increasing values, particularly after 1994, with episodic declines in 1979 and 1993. Sugar cane production (1961 to 2006) has declined sharply from 1961 to 1980 and remained relatively stable from 1980 to 2006.

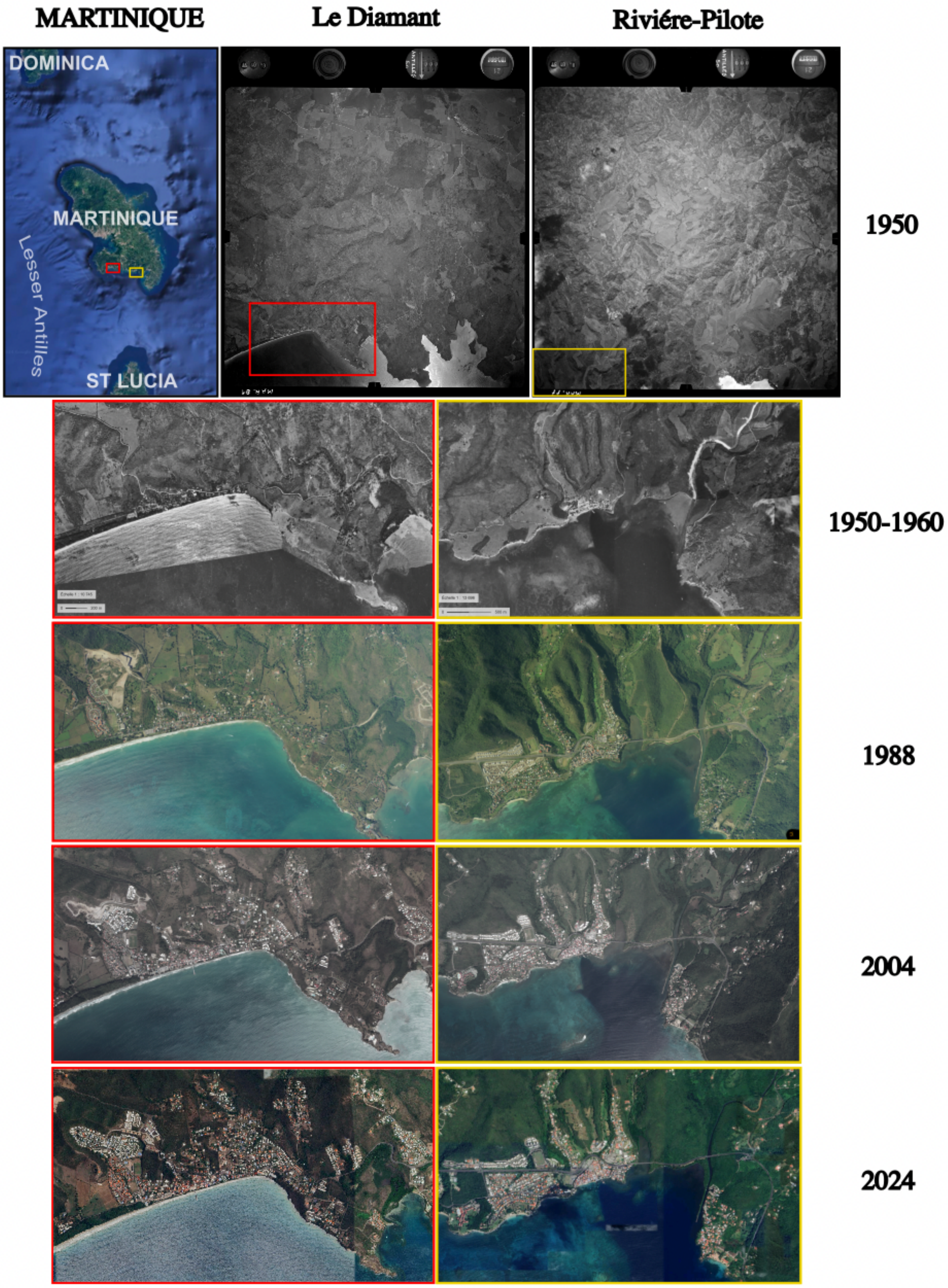

Fig. S8 Aerial footage and satellite images showing the evolution of land uses in Southern Martinique in 1950, 1988, 2004, and 2024 from https://remonterletemps.ign.fr/telecharger/, https://www.geoportail.gouv.fr/carte, and https://www.maps.google.com (accessed in 2024). Agriculture and deforestation appear to dominate land use changes between 1950 and the late 1980s, while the expansion of coastal urbanization can be seen in more recent decades.

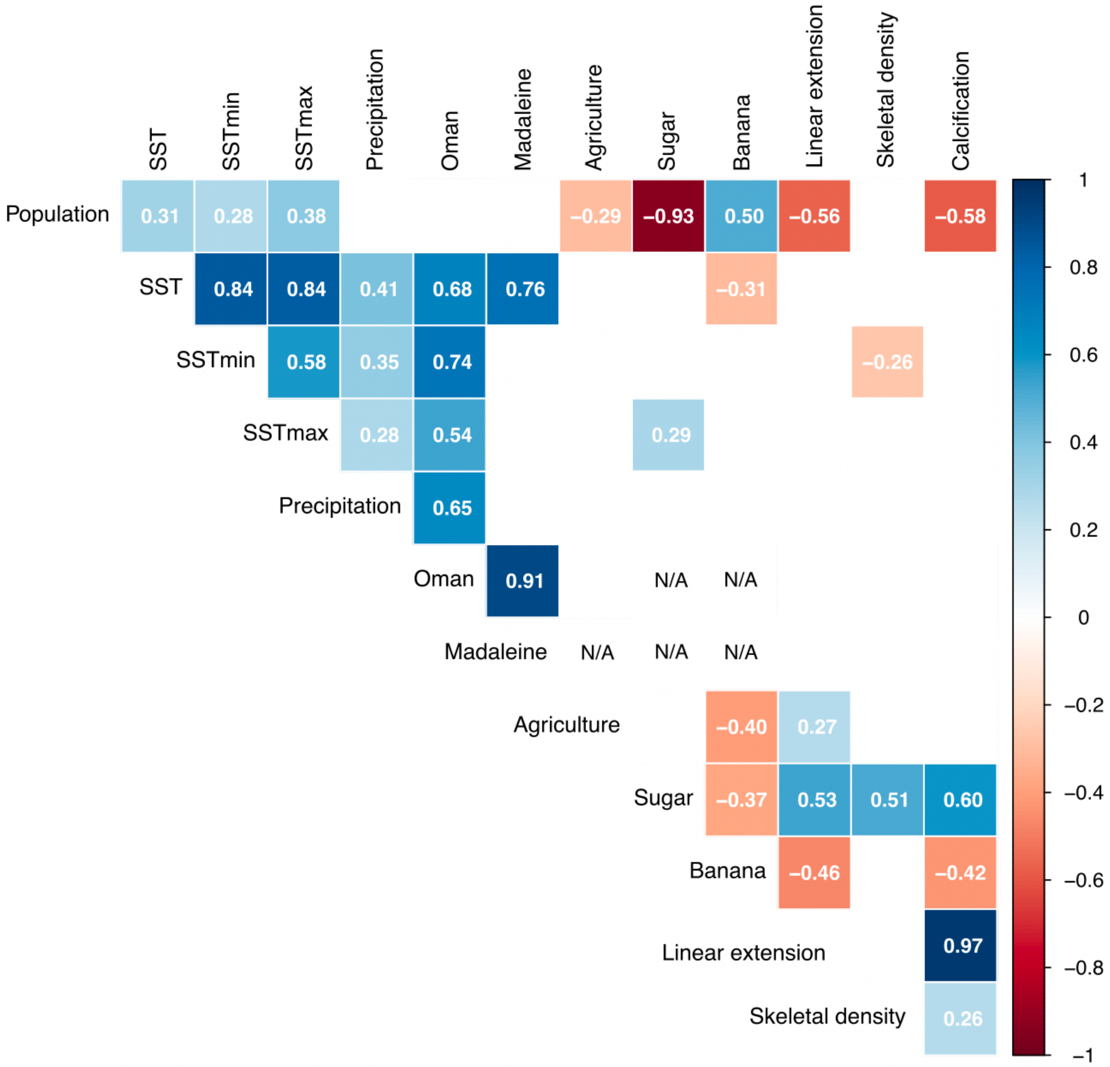

Fig. S9 Pearson correlation matrix between log-transformed annual values of linear extension (1950 to 2020, n = 71), skeletal density (1950 to 2020, n = 71), and calcification rate (1950 to 2020, n = 71), mean sea surface temperature (SST, 1950 to 2020, n = 71), minima SST (SST_{min}, 1950 to 2020, n = 71), maxima SST (SST_{max}, 1950 to 2020, n = 71), precipitation (1950 to 2020, n = 71), agricultural area (1950 to 2020, n = 60), banana production (1961 to 2006, n = 47), sugar cane production (1961 to 2006, n = 47), and river discharge from Oman (1995 to 2020, n = 20) and Madeleine (2012 to 2020, n = 9). Pearson coefficients are presented inside the boxes with significant correlations ($p < 0.05$) highlighted in colors (red = negative, blue = positive). Blank boxes represent non-significant correlations, and N/A represents correlations that were not applicable due to the lack of overlapping data.

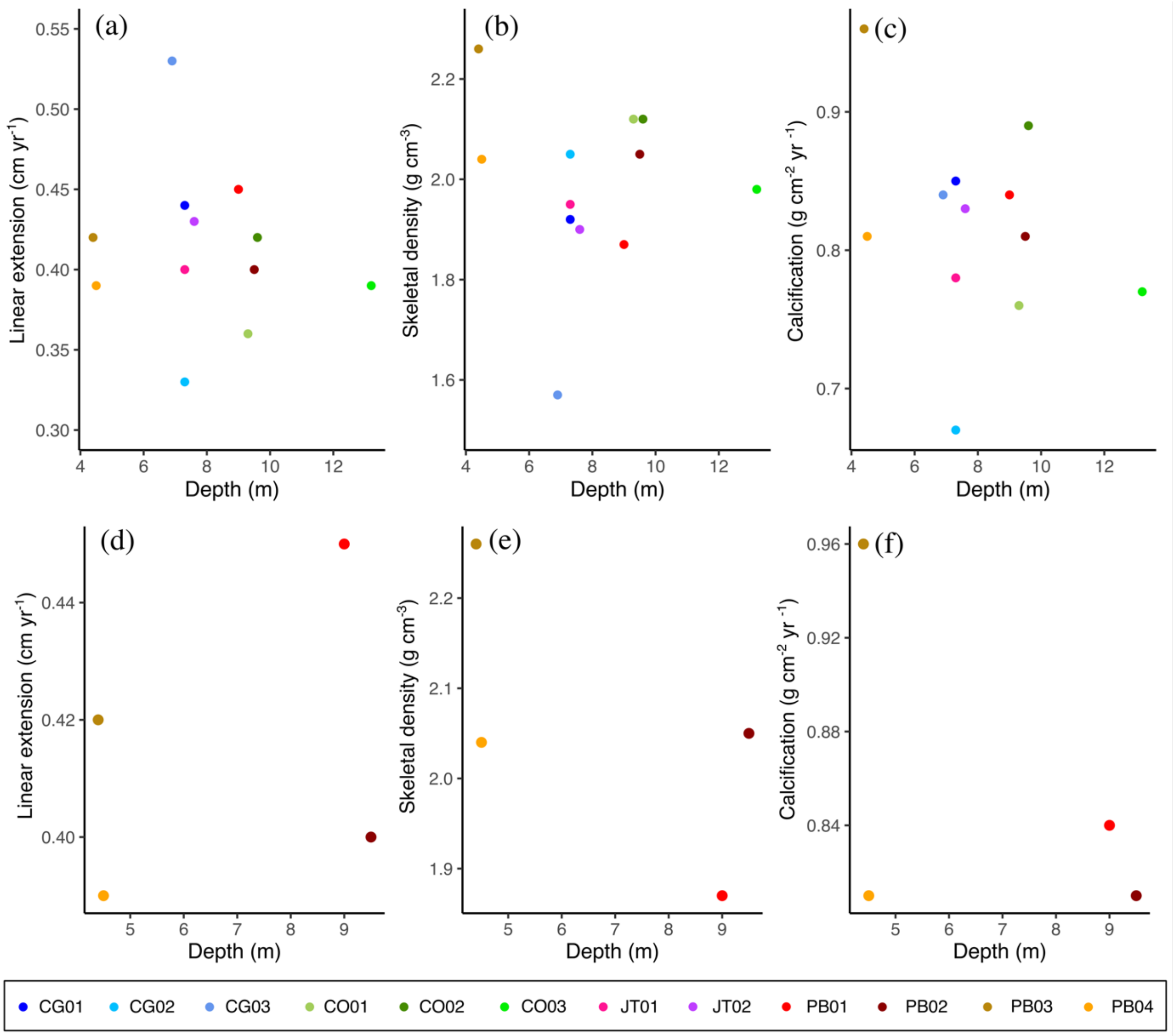

Fig. S10 Scatter plots based on annual time series of growth parameters and depth. Circles represent the mean of each parameter and colors show from which coral these parameters were obtained. Coral growth records from all sites are shown in (a), (b), and (c). Coral records from Point Borgnèse reef (d, e, and f) highlight the lack of growth differences across depths. Since no statistical significance ($p < 0.05$) exists between growth parameters and depth, no regression lines (95% confidence interval) and the corresponding coefficient of determination (r^2) and p-value were indicated.

References

1 Helmle, K., Kohler, K. & Dodge, R. Relative optical densitometry and the coral X-radiograph densitometry system: Coral XDS. *International Society Reef Studies, European Meeting Cambridge, England.* (2002).

2 Stearn, C. W., Scoffin, T. P. & Martindale, W. Calcium carbonate budget of a fringing reef on the west coast of Barbados. Part I-zonation and productivity. *Bull Mar Sci* **27**, 479-510 (1977).

3 Hubbard, D. K. & Scaturo, D. Growth rates of seven species of scleractinian corals from Cane Bay and Salt River, St. Croix, USVI. *Bull Mar Sci* **36**, 325-338 (1985).

4 Huston, M. Variation in coral growth rates with depth at Discovery Bay, Jamaica. *Coral Reefs* **4**, 19-25 (1985).

5 Guzmán, H. M., Jackson, J. B. C. & Weil, E. Short-term ecological consequences of a major oil spill on Panamanian subtidal reef corals. *Coral Reefs* **10**, 1-12 (1991).

6 Guzmán, H. M. & Tudhope, A. W. Seasonal variation in skeletal extension rate and stable isotopic ($^{13}C/^{12}C$ and $^{18}O/^{16}O$) composition in response to several environmental variables in the Caribbean reef coral *Siderastrea siderea*. *Mar Ecol Prog Ser* **166**, 109-118 (1998).

7 Torres, J. L. & Morelock, J. Effect of terrigenous sediment influx on coral cover and linear extension rates of three Caribbean massive coral species. *Caribb J Sci* **38**, 222-229 (2002).

8 Carricart-Ganivet, J., Vásquez-Bedoya, L., Cabanillas-Terán, N. & Blanchon, P. Gender-related differences in the apparent timing of skeletal density bands in the reef-building coral *Siderastrea siderea*. *Coral Reefs* **32**, 769-777 (2013).

9 DeLong, K. L. et al. A reconstruction of sea surface temperature variability in the southeastern Gulf of Mexico from 1734 to 2008 C.E. using cross-dated Sr/Ca records from the coral *Siderastrea siderea*. *Paleoceanography* **29**, 403-422 (2014).

10 Flannery, J. A., Richey, J. N., Thirumalai, K., Poore, R. Z. & DeLong, K. L. Multi-species coral Sr/Ca-based sea-surface temperature reconstruction using *Orbicella faveolata* and *Siderastrea siderea* from the Florida Straits. *Palaeogeogr Palaeoclimatol Palaeoecol* **466**, 100-109 (2017).

11 Rippe, J. P. et al. Corals sustain growth but not skeletal density across the Florida Keys Reef Tract despite ongoing warming. *Glob Chang Biol* **24**, 5205-5217 (2018).
